# Supplementary material for: Structural mechanisms for binding and activation of a contact-quenched fluorophore by RhoBAST
Source: Nat Commun. 2024 May 17;15:4206. doi: 10.1038/s41467-024-48478-9 (PMC11101630; doi:10.1038/s41467-024-48478-9)
Supplement: Supplementary file 1 — Supplementary information [file 41467_2024_48478_MOESM1_ESM.pdf]

Supplementary Information

Structural mechanisms for binding and activation of a contact-quenched fluorophore by RhoBAST

Yufan Zhang[1, 5], Zhonghe Xu[2, 5], Yu Xiao[2, 5], Haodong Jiang[3, 5], Xiaobing Zuo[4], Xing Li[3, *], Xianyang Fang[1, 2, *]

[1]Key Laboratory of RNA Science and Engineering, Institute of Biophysics, Chinese Academy of Sciences, Beijing 100101, China

[2]Beijing Frontier Research Center for Biological Structure, School of Life Sciences, Tsinghua University, Beijing 100084, China

[3]Beijing Institutes of Life Science, Chinese Academy of Sciences, Beijing, 100101, China

[4]X-ray Science Division, Argonne National Laboratory, Lemont IL 60439, USA

[5]These authors contributed equally: Yufang Zhang, Zhonghe Xu, Yu Xiao, Haodong Jiang

Content

Supplementary Methods......3

Supplementary Figure 1 9

Supplementary Figure 2 10

Supplementary Figure 3 11

Supplementary Figure 4 12

Supplementary Figure 5 13

Supplementary Figure 6 15

Supplementary Figure 7 16

Supplementary Figure 8 17

Supplementary Figure 9 18

Supplementary Figure 10 19

Supplementary Table 1 20

Supplementary Table 2 22

Supplementary Table 3 23

Supplementary Table 4 23

Supplementary Table 5 24

Supplementary Table 6 25

Supplementary Table 7 25

Supplementary Table 8 26

Supplementary Table 9 26

Supplementary Table 10 27

Supplementary References......28

Supplementary Methods:

Quantum Chemical Calculations

If not explicitly stated otherwise, we employed r^2scan-3c method to performed geometrical optimization using ORCA 5.0.4 program with implicit solvent model (CPCM)[1-3]. r^2scan-3c recently developed by Grimme group incorporates modification of basis set and dispersion, as well as basis set superposition error correction (3c)[3]. Such 3c correction is important for reliable description of the noncovalent interactions which play essential roles in the formation of self-stacking configuration. It has been demonstrated that the computation using r^2scan-3c could achieve well performance with affordable cost on conformational energies as well as non-covalent interactions[3]. The frequency calculation was also performed to ensure that the obtained geometry locates at the minimal of potential energy surface.

Single point energy calculations for the representative conformations of TMR-DN were carried out using double-hybrid density functional PWBP95-D3 and ωB97x-2-D3 with larger basis set, def2-QZVPP in SMD implicit solvent model[4-6]. We leveraged resolution of the identity (RI) approximation methods to accelerate all DFT computations in ORCA.

Molecular Dynamics simulations

Preparation of initial models for MD simulation. For TMR-DN alone system, we prepared two different initial configurations for simulations. The first one was directly taken from the crystal structure, which resembled to DN-phenyl stacked configuration. The second one was extended conformer constructed using GaussView 6 program. It was further optimized using r^2scan-3c method combined with implicit solvent model (CPCM)[2].

Additionally, the frequency calculation was also performed to ensure that the obtained geometry locates at the minimal of potential energy surface.

For RhoBAST-TMR-DN complex system, we also constructed two initial models for simulations. The crystal structure with complete TMR-DN was used by removing U1A protein and substituting the U1A loop with GAAA tetraloop using FARFAR2. To reduce computational cost, we further deleted the first three base-pairs of P1 stem, and mutated the terminal base pair A-U to G-C pair. This deleted fragment is far from binding pocket for TMR-DN, and is presumed to have minor effect on binding of TMR-DN to RNA. Two Mg^{2+} ions in bound form in the crystal structure were reserved. Considering that the DN and linker moieties from the other two complexes in the asymmetrical unit of crystal structure are missing due to poor electron density, we construct a second initial model by substituting the TMR-DN with our QM-optimized one (extended). The structural differences between the two initial models in bound form lie in the linker as well as DN.

Reparameterization of TMR-DN. The QM-optimized TMR-DN (extended configuration) was used for restrained electrostatic potential charges 2 (RESP2) fitting[7]. Firstly, we employed Gaussian 09E program to calculate the Merz-Kollman electrostatic potential (ESP) at PBE0 level with ma-TZVPP basis set in both of aqueous (IEFPCM model) and gas phases[8-10]. Then the obtained ESP files were fed into Antechamber module implemented in Amber22 package for regular RESP fitting[11]. The RESP2 charge is a weighted average of aqueous- and gas-charge (60% aqueous, 40% gas). The RESP2 is regarded as a more accurate and robust method, compared to the original RESP methods which is developed in thirty years ago and leverages the fortuitous over polarization of the HF/6-31G(d) in gas phase. The other force field parameters for TMR-DN including atom

types and bonded parameters are automatically assigned by Antechamber module using General Amber Force Field 2 (gaff2)[12], which is compatible to Amber force filed of biomolecules. The force field parameters file of TMR-DN in GROMACS format is provided as Supplementary Data 1.

Simulation settings. As mentioned above, the parameters for TMR-DN were generated using the Antechamber. The RhoBAST RNA was described by parmbsc0+χOL with van der Waals radii correction to phosphate oxygen atoms[13-17], and water was parameterized using the 4-point optimal point-charge (OPC) model[18]. The ion parameters compatible to OPC water developed by the Merz group were employed in our simulations[19]. The rhombic dodecahedron boxes were adopted in our simulation, ensuring the solute with at least 10 Å distal to box face. For complex system, KCl was added to neutralize the system and yielded an ionic concentration of 150 mM. TMR-DN alone system contains 2754 water. The final system of RhoBAST-TMR-DN complex comprise 47244 atoms, including 11363 water, 2 bound-form Mg^{2+}, 76 K^+ and 32 Cl^- ions.

All MD simulations were performed using GROMACS 2020 or 2022 patched by PLUMED2.9[20,21]. All systems were firstly minimized, followed by a step-wise heating from 10 K to 300 K. Two sequential NPT simulations (T = 300 K and P = 1 bar) were then carried out. During the heating as well as NPT phases, the positional restraints imposed on RNA as well as ligand and Mg^{2+} were gradually removed. Finally, for each system, the production run was carried out in NPT ensemble without any restraints for at least 50 ns. For TMR alone system, we performed two independent simulations with different initial starting structure. For complex system, we performed two sets of simulations with different starting model. Each set of simulations comprises two replicas with different random seed.

The temperature and pressure were maintained by the velocity-rescaling thermostat, and the Parrinello-Rahman barostat respectively[22,23]. The covalent bonds involved by hydrogen atoms were constrained using the LINCS algorithm[24], allowing an integration time step of 2 fs. Periodic boundary conditions were applied in three directions. van der Waals and short-range electrostatic interactions were calculated at a cutoff distance of 10 Å, whereas long-range electrostatic interactions were computed by the particle mesh Ewald method[25].

We can not obtain the converged statistics through conventional MD simulations within 100-ns for complex system. Thus, we resorted to enhanced-sampling methods. Herein, we employed a modified version of replica exchange with solute tempering (REST2) combined with tempering parallel, which referred to as REHT[26]. REST2 is one of Hamiltonian replica exchange methods, which selectively tempers a given region (hot region) by means of scaling the potential energy of hot region, and keep temperature of all replica constant. While the REHT couples tempering parallel with scaling of the potential energy, allowing it to cross energy barriers rapidly.

In our simulations of TMR-DN alone system, 20 independent replicas were run. TMR-DN (91 atoms) was treated as hot region with scaled factors spanning 1.0 to 0.5 following geometrical distribution. While the bath temperatures for all replicas are spanning 300 to 360 K. In our simulations of complex system, 32 independent replicas were adopted. We selected the linker and the quencher (40 atoms) as hot region with scaled factors spanning 1.0 to 0.25, leaving the energy functions of TMR moiety unperturbed in our all replicas. And the bath temperatures for REHT simulation of complex are spanning 300 to 350 K. For both systems, the exchanges attempt between neighbor replica is set to 10 ps, and the averaged acceptance probability was around 0.32 ~ 0.33. In our REHT simulations, we

adopted NVT ensemble, and prepared different initial configurations for each replica, which were taken from our conventional MD simulations. Each replica was carried out up to 200 ns, totaling 4 μs and 6.4 μs of simulation time for TMR-DN alone and complex, respectively. The conformers from the replica running at the lowest temperature were used for analysis.

Analysis of MD simulation trajectory

To visualize distribution of DN around TMR or RNA, we performed Spatial Distribution Function (SDF) analysis for DN centroid. Firstly, we performed structural alignment using xanthene (for TMR-DN) or partial RNA fragment (pocket and neighboring nucleotide: nt 30 - 58 in crystal structure) as reference for whole ensemble, to remove the translation and rotation of system. Secondly, the coordinates of the TMR or TMR plus RNA as well as centroid of DN ring were save as PDB file in VMD[27] using in-house script. Then, the PDB files were converted to binary format of coordinate (i. e. dcd) to reducing file size. Finally, we utilized VolMap plugin in VMD to perform SDF analysis, and render the pictures with VMD.

To classify the conformer ensemble from MD simulation, we analyzed the structural features including contact number, stacking interaction and the distance between centers of aromatic rings (centroid distance) in VMD using in-house script.

We evaluated the contact between different moieties (i. e. xanthene or phenyl rings, TMR, quencher) with a continuous function, which is adapted from the previous work of Best[28]:

$$Q(I,J) = \sum_{i}\sum_{j}[1 + \exp(\beta \times (R_{i,j} - \lambda R_0))]^{-1}$$

Where the β is a smooth parameter set to be 5 $Å^{-1}$; $R_{i,j}$ is the non-hydrogen atomic-pair distance between different moieties *I* and *J*; both of the factor λ and contact distance cutoff R_0 control the formation of contact, which are taken to be 1.5 and 3.0 Å. When atomic pair is separated by 3.5, 4.5 (equal to the product of λ and R_0) and 5.0 Å, the contact number is 0.99, 0.50 and 0.08, respectively.

The stacking interaction of quencher on xanthene or phenyl was characterized using the geometrical setup as Supplementary Figure 10. The reference Cartesian frame of xanthene or phenyl ring was defined as following. The origin of frame is the geometrical center of the ring. The x-axis is coaxial with the rotatable bond connecting xanthene and phenyl, which is also through the centroid of ring. Whereas the y-axis is within the respective plane and forms 90 degree with x-axis. The z-axis is thus the normal to the plane. The mutual orientation angle (θ) was calculated as the angle formed by normal of two rings. The position of quencher (centroid) with respect to reference frame is determined through 3 translational parameters (Δx shift, Δy slide and Δz rise). The two rings were identified as stacking based on 4 conditions. Their mutual angle θ should be less than 45 degree and larger than 135 degree. Their 3 translation parameters also should fall in a certain region, as presented in Supplementary Figure 10. More specifically, the Δz-rise cutoff for both of xanthene and phenyl is set to ± 4.0 Å, in consistent with previous work[29]. Whereas the Δx-slide cutoff for both of xanthene and phenyl is set to ± 1.6 Å, comparable to six-membrane ring. The Δy-shift cutoff for xanthene and phenyl is set to ± 5.0 and ± 1.6 Å, respectively.

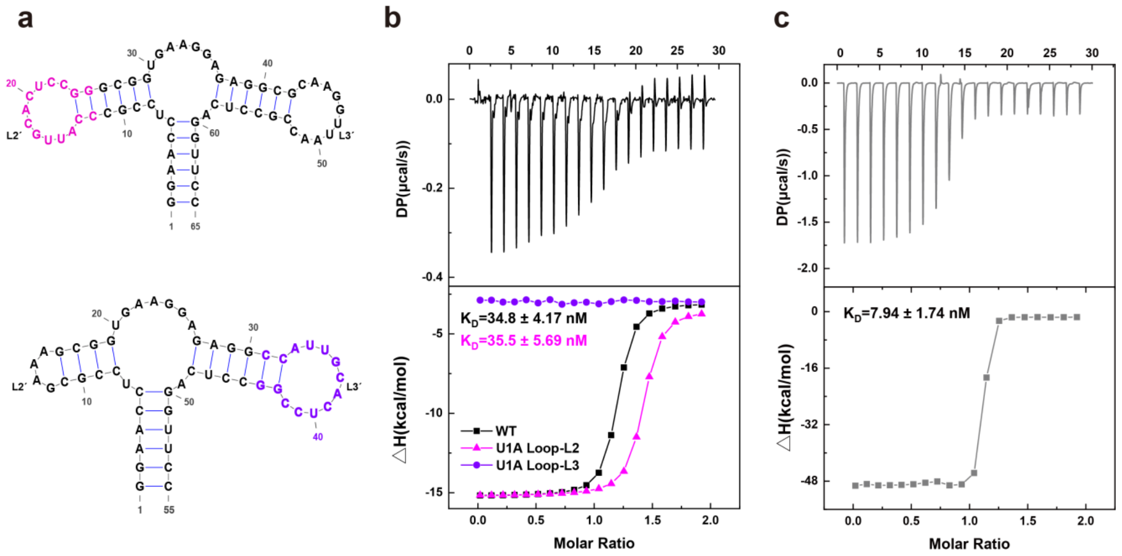

Supplementary Figure 1 | Characterization of the RhoBAST-U1A loop complex. a, Engineering the U1A loop into L2´ (magenta) or L3´ (purple) of the RhoBAST aptamer. **b,** Binding of TMR-DN to the RhoBAST or its mutants containing the U1A Loop as measured by ITC assay. **c,** Binding of U1A protein to the RhoBAST-U1A Loop-L2 by ITC assay. Source data for panels **b-c** are provided as a Source Data file.

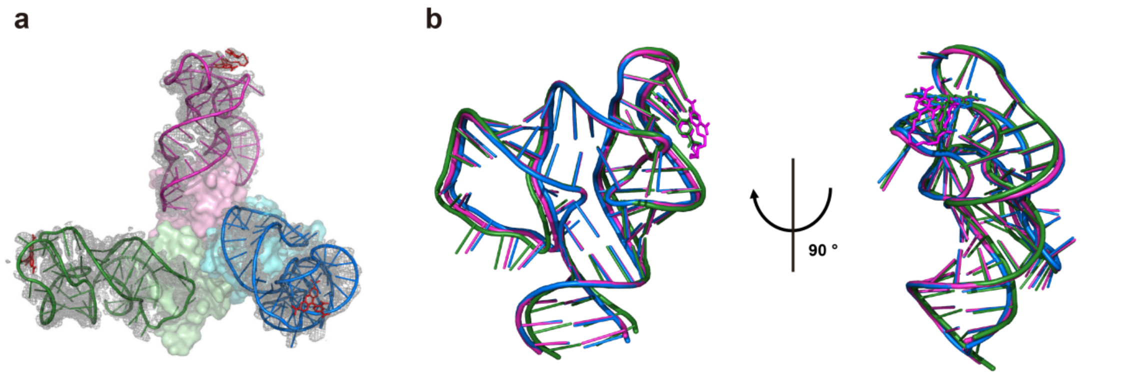

Supplementary Figure 2 | The initial phase was obtained by molecular replacement using the U1A protein as a search model. a, Each asymmetrical unit consists of three complexes in the Crystalline. The U1A proteins are shown as surface representation (Type of map: 2Fo-Fc, contour level: sigma=1.0). **b,** Structural comparison of the three RNA structures from one asymmetrical unit in the crystal lattice.

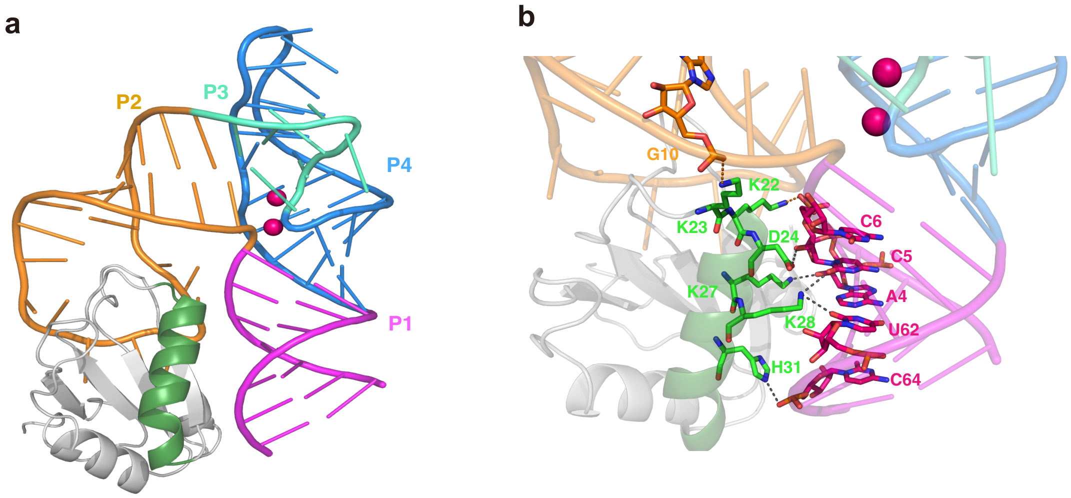

Supplementary Figure 3 | The non-specific interaction between U1A protein and RhoBAST. a, Overall structure of RhoBAST in complex with U1A protein, where the helix 2 engaged in non-specific interaction with RNA is highlighted in green. **b,** The non-specific interactions of U1A protein with duplexes of P1 and P2 of RNA through hydrogen bonds and salt bridges, which are shown in gray and orange dashed lines, respectively.

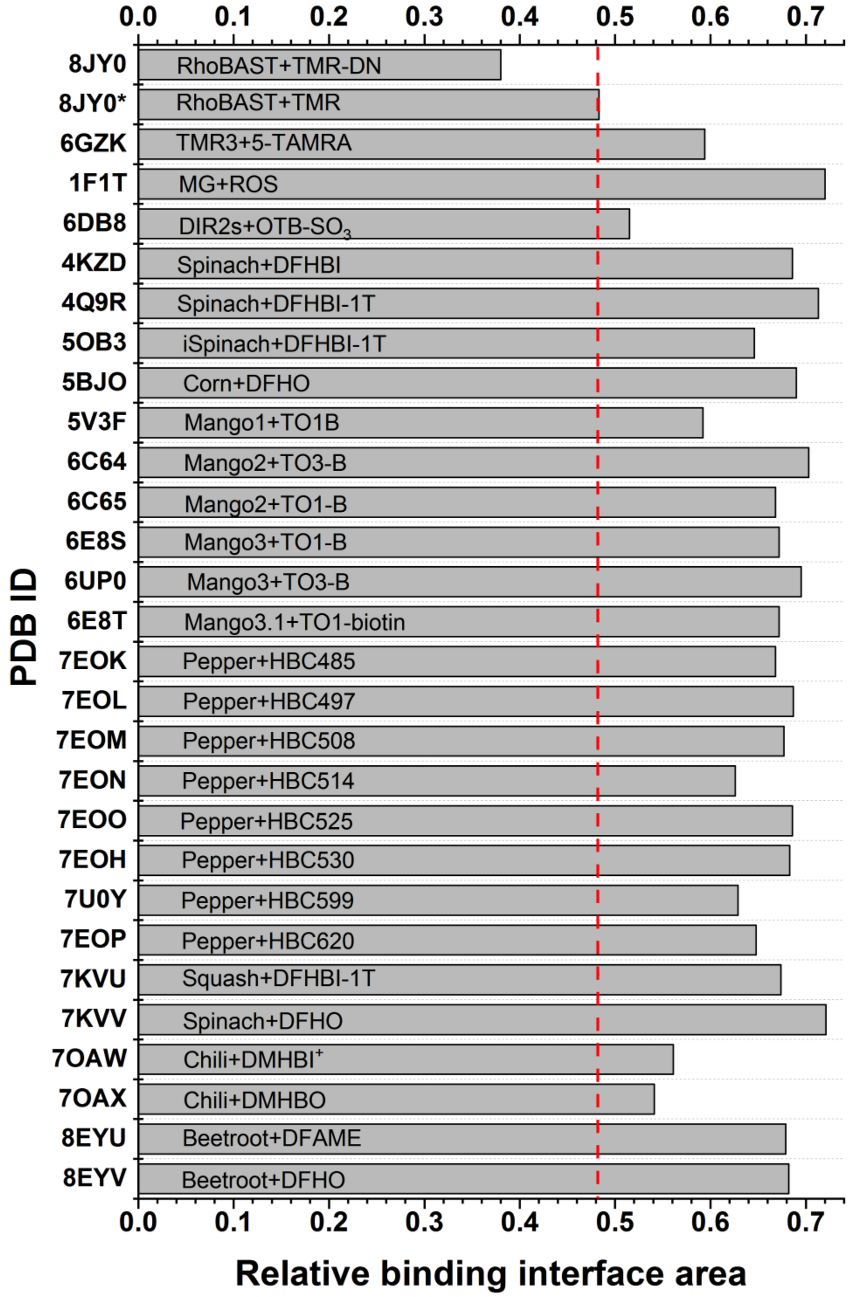

Supplementary Figure 4 | Comparison of the relative binding interface area among different aptamer-ligand pairs. The relative binding interface area is defined as the ratio of binding interface to the $SASA_{ligand}$ in Supplementary Table 2. 8JY0* represents the two other structures in the asymmetrical unit of 8JY0, of which the electron density for DN quencher and linker is poor. According to previous work, TMR exhibit similar binding affinity to RhoBAST as TMR-DN. The red dash line corresponds to the relative binding interface area value of RhoBAST-TMR. Source data is provided as a Source Data file.

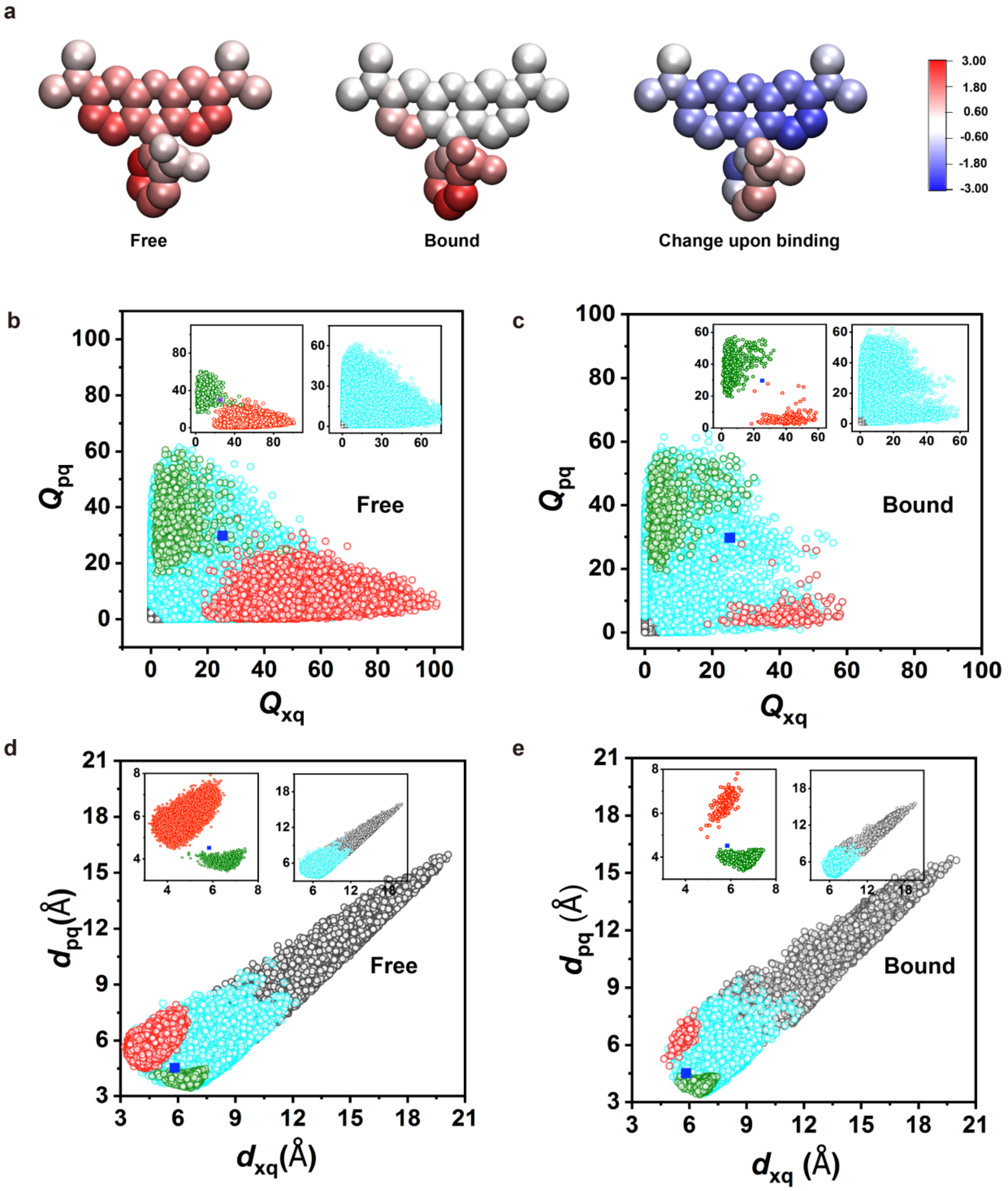

Supplementary Figure 5 | 2D scatter plots of Q_{xq} vs Q_{pq} and d_{xq} vs d_{pq} of the TMR-DN alone and in complex with RhoBAST. a, Color visualization of contact number for the atom of TMR, which characterize as contact interaction between atom of TMR and DN fragment rather than total contact numbers between fragments. The values or the changed values upon binding to RNA are mapped to the atoms of TMR, which are colored in blue-white-red spectrum. **b, c,** The contact number (Q) between the DN quencher and the xanthene (Q_{xq}) or phenyl ring (Q_{pq}) for conformer in the free (b) and bound-form (c). **d, e,**

The centroid distances between the xanthene or phenyl ring and DN, which are denoted as d_{xq} and d_{pq}, for conformer in free (d) and bound-form (e). The DN-xanthene stacked, DN-phenyl stacked, contact-but-unstacked, and non-contact class of conformers are colored red, green, cyan and gray, respectively. While the bound-form conformer from the crystal structure is indicated with the blue square. Source data for panels **b-e** are provided as a Source Data file.

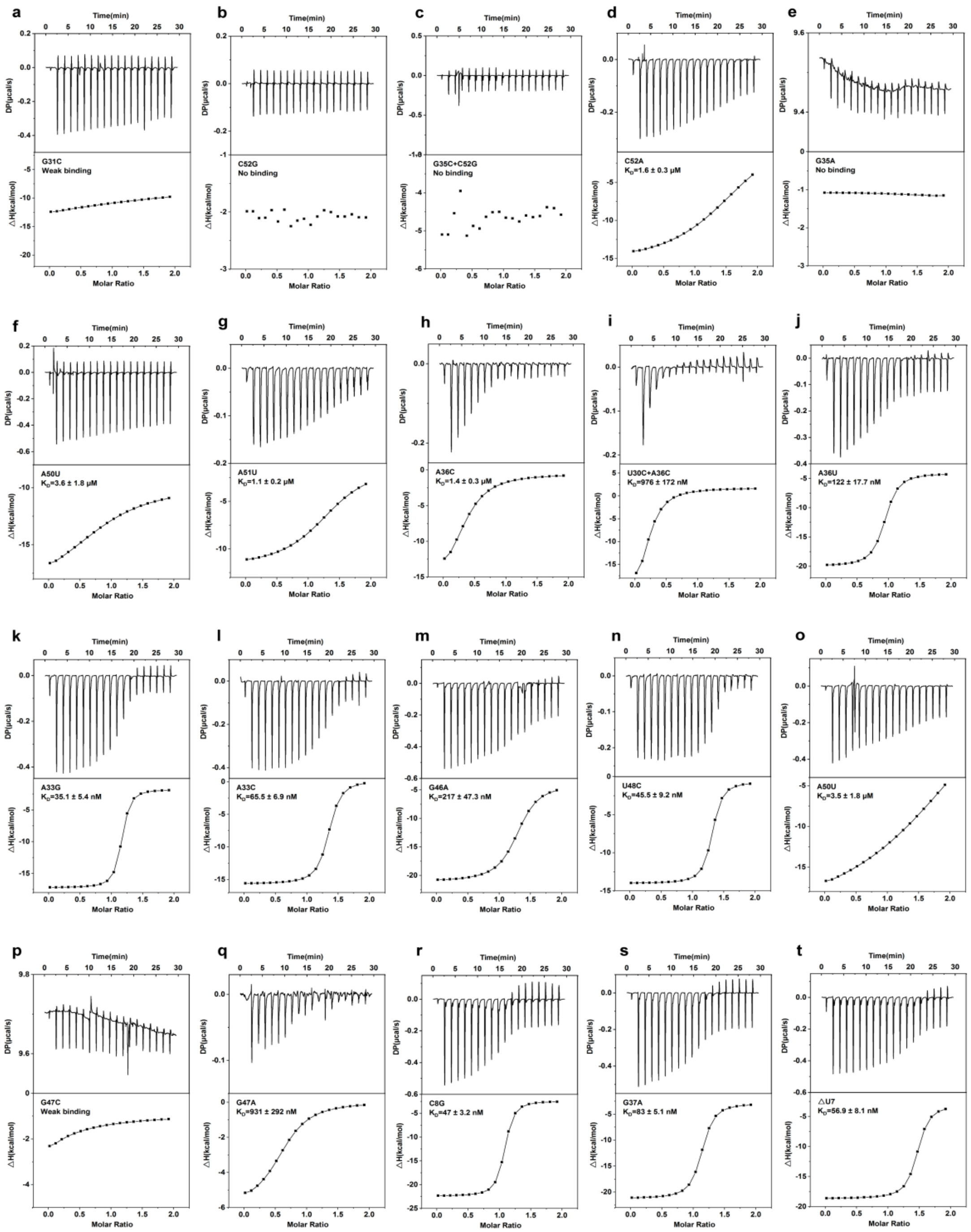

Supplementary Figure 6 | ITC analysis of the mutational effects on the binding between RhoBAST and TMR-DN. a-t, The ITC curve and binding parameter for each mutant, including G31C, C52G, G35+C52G, C52A, G35A, A50U, A51U, A36C, U30C+A36C, A36U, A33G, A33C, G46A, U48C, A50U, G47C, G47A, C8G and ΔU7.

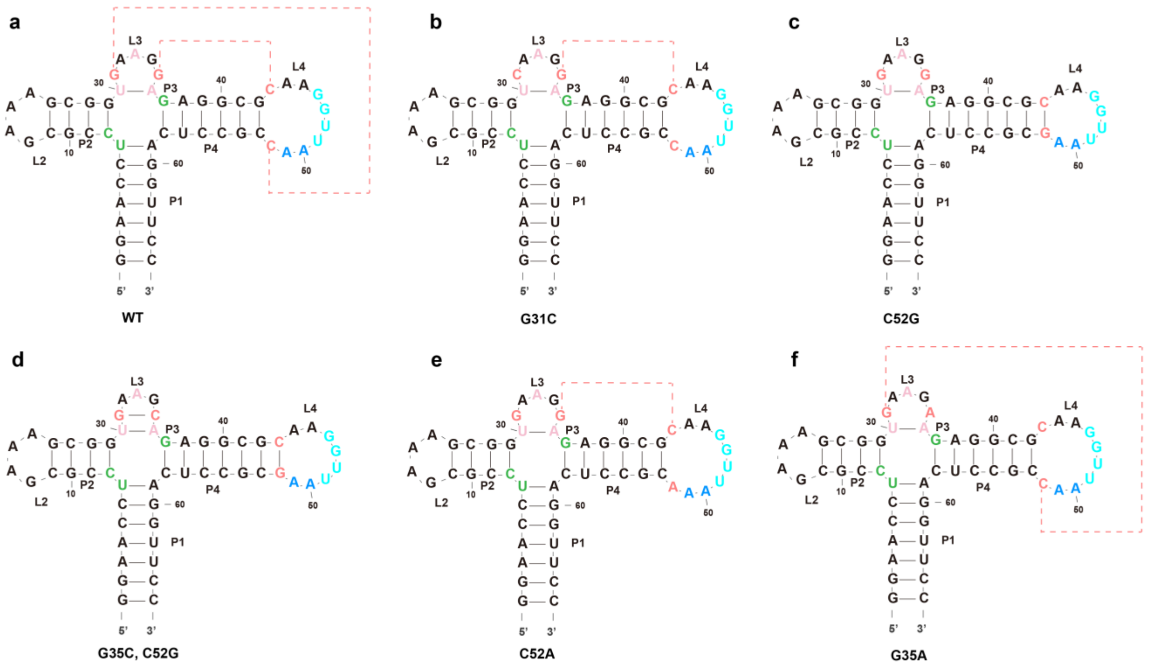

Supplementary Figure 7 | The second structure of the mutants which involved in the interwoven WC base pairs. a, The secondary structure of RhoBAST wild type (WT). **b-f,** Predicted secondary structures of the different RhoBAST mutants. The color code for the nucleotides involved in mutations is the same as Fig. 5a in the main text.

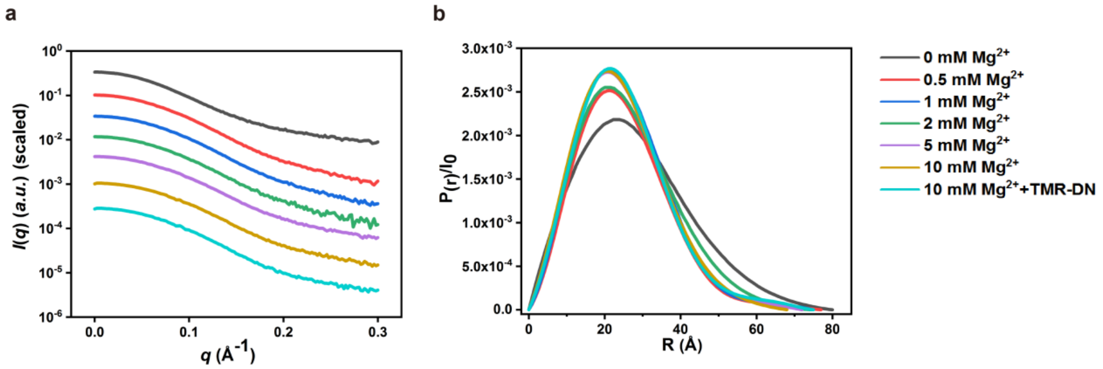

Supplementary Figure 8 | The effects of Mg^{2+} on the folding of RhoBAST analyzed by SAXS. a, b, The scattering profiles (a) and the PDDFs (b) of RhoBAST wild type in the presence of different Mg^{2+} concentrations or both TMR-DN and 10 mM Mg^{2+}. Source data is provided as a Source Data file.

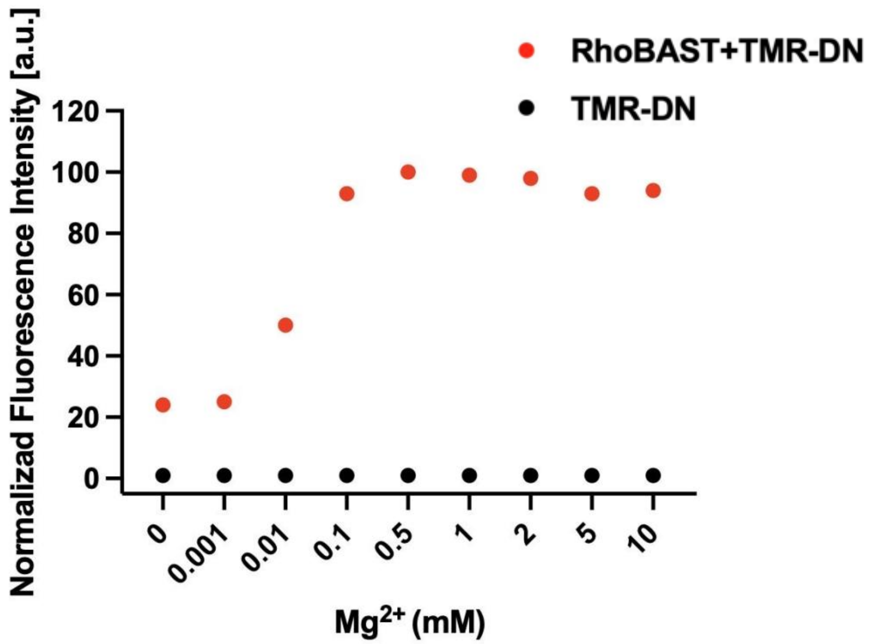

Supplementary Figure 9 | Mg^{2+}-dependence of the fluorescence emmission of TMR-DN alone and in the presence of RhoBAST. Mg^{2+} is essential for RhoBAST-TMR-DN complex formation. The fluorescence of TMR-DN alone and the RhoBAST-TMR-DN complex were determined in a buffer containing 20 mM Tris (pH 7.5), 100 mM KCl and various concentrations of $MgCl_2$ (1 µM – 10 mM). Source data is provided as a Source Data file.

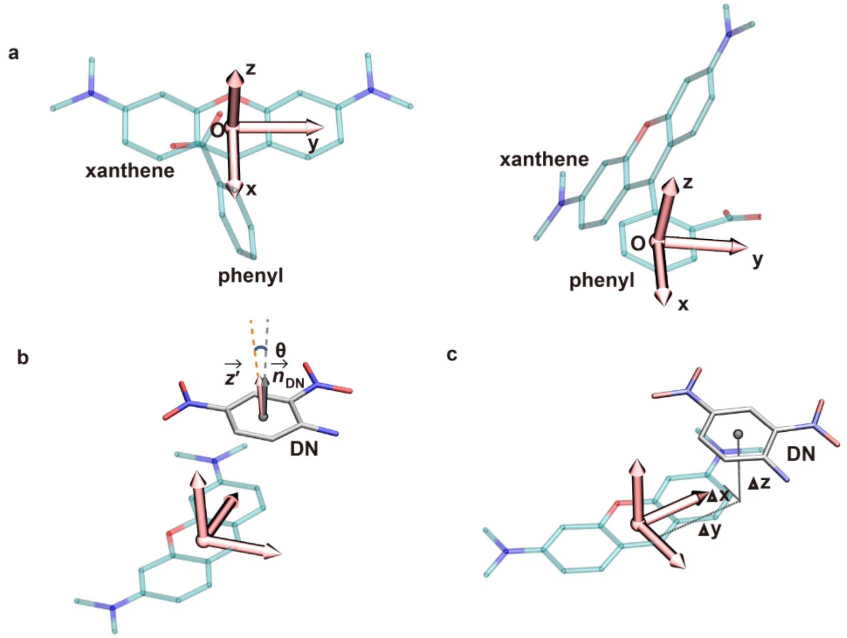

Supplementary Figure 10 | Schematic representation of geometrical parameters to identify stacking interactions between xanthene and DN or phenyl and DN. a, Definition of the reference Cartesian frame on the xanthene (left) and phenyl ring (right). The origin is the centroid of ring. The x-axis is co-axial with the rotatable bond connecting xanthene and phenyl ring. The y-axis is within the respective plane and forms 90 degree with x-axis. The z-axis is cross product between x-axis and y-axis. **b**, Definition of mutual orientation angle which is determined by the angle between normal of two rings. **c**, Three translation parameters including Δx, Δy and Δz, which are dot products between the vector point from the origin to the centroid of DN (gray sphere) and the respective standard vector (i.e. $\vec{x}$, $\vec{y}$, $\vec{z}$).

Supplementary Table 1. The data collection and refinement statistics.

Data set	RhoBAST-TMR-DN
**PDB code**	8JY0
**Data collection**	
Beamline	SSRF BL19U1
Wavelength (Å)	0.979
Resolution (Å)	50-2.75 (2.80 - 2.75)
Space group	$I\,2_1\,2_1\,2_1$
Cell dimensions	108.5 158.9 182.4<br>90.0 90.0 90.0
Unique reflections	40161 (1927)
Completeness (%)	97.4 (95.8)
R_{meas} (%)	10.5 (100.4)
R_{pim} (%)	4.3 (43.6)
$CC_{1/2}$ (%)	99.7 (67.9)
Redundancy	5.4 (4.9)
$I/\sigma(I)$	15.6 (1.3)
**Refinement**	
R_{work} (%)	20.2 (32.7)
R_{free} (%)	23.1 (35.6)
**R.m.s.d.**	
Bond lengths (Å)	0.117
Bond angles (°)	1.89
**No. of atoms**	6864
RNA/protein	6559
Ligand/ion	121
water	100
**Average B factors (Å^2)**	56.57
RNA/protein	55.96
Ligand/ion	76.37
water	47.47
**Ramachandran plot (%)**	

Favored region	97.66
Allowed region	2.34
Outliers	0.00

$R_{meas} = \Sigma_h\sqrt{[n/(n-1)]}\Sigma_i|I_{h,i} - <I_h>|/\Sigma_h\Sigma_i I_{h,i}$, $R_{pim} = \Sigma_h\sqrt{[1/(n-1)]}\Sigma_i|I_{h,i} - <I_h>|/\Sigma_h\Sigma_i I_{h,i}$, where I_h is the mean intensity of the *i* obervations of symmetry related reflections of *h*. $R_{factor} = \Sigma|F_{obs} - F_{calc}|/\Sigma F_{obs}$, where $F_{obs} = F_P$, and F_{calc} is the calculated protein structure factor from the atomic model. R_{work} was calculated with 95.0% of the reflections. R_{free} was calculated with 5.0% of the reflections which were randomly selected and did not used for structure refinement. R.m.s.d. (root-mean-square error) in bond lengths and angles are the deviations from ideal values. Values in parentheses correspond to the last resolution shell. HKL2000 and Phenix. refine calculated the statistics of data collection and refinement, respectively.

Supplementary Table 2. The solvent accessible surface areas of RhoBAST and its relevant aptamers in complex with their cognate ligands, related to Fig. 7d and Supplementary Figure 4.

PDB ID	$SASA_{RNA}$ (Å^2)	$SASA_{ligand}$ (Å^2)	$SASA_{complex}$ (Å^2)	Binding interface (Å^2)	Relative binding interface
8JY0	12225.1	860.9	12431.8	327.1	0.380
8JY0 (TMR)#	12225.1	586.9	12245.3	283.4	0.483
8JY0 (xanthene)#	12225.1	454.2	12181.6	248.9	0.548
6GZK[30]	8709.2	637.3	8588.8	378.8	0.594
6GZK (xanthene)#	8709.2	463	8601.6	285.3	0.616
1F1T[31]	7000.1	560.5	6753.8	403.4	0.720
1F1T (xanthene)#	7000.1	458.3	6803.3	327.5	0.715
6DB8[32]	10729.4	584.2	10711.7	300.9	0.515
6DB8 (OTB)#	10729.4	469.2	10707.4	245.6	0.523
4KZD[33]	15205.8	432.1	15045.4	296.3	0.686
4Q9R[33]	15121.1	497.7	14911.5	353.6	0.711
5BJO[34]	11140.7	464.8	10964	320.7	0.69
5OB3[35]	12475.7	405.2	12357.5	261.7	0.646
5V3F[36]	5897.3	947.1	5723.2	560.6	0.592
6C64[37]	6525.8	479.8	6331.1	337.2	0.703
6C65[37]	6469.2	623.5	6259.7	416.4	0.668
6E8S[38]	6520.8	645.2	6299.4	433.3	0.672
6E8T[38]	6229.4	527.8	6047.7	354.8	0.672
6UP0[39]	6450.8	542.5	6238.8	377.3	0.695
7EOH[40]	8621.8	540	8424.6	368.6	0.683
7EOL[40]	8703.6	529.9	8505.2	364.1	0.687
7EOK[40]	8589.7	584.1	8393.5	390.1	0.668
7EOM[40]	8576.6	533.5	8388.1	361	0.677
7EON[40]	8616.1	608.7	8462.2	381.2	0.626
7EOO[40]	8618	545	8414.7	374.2	0.686
7EOP[40]	8618	582.2	8445.8	377.2	0.648
7KVU[41]	13903.7	505.1	13728.1	340.4	0.674
7KVV[41]	13913.1	471.7	13704.8	340	0.721
7OAW[42]	9527.4	611.1	9453.4	342.5	0.561
7OAX[42]	9500.3	647.9	9447.4	350.4	0.541
7U0Y[40]	11884.5	584.5	11733.9	367.6	0.629
8EYU[43]	8857.5	513.5	8673.6	348.6	0.679
8EYV[43]	8292.9	451.4	8128.8	307.8	0.682

For detailed comparison of RhoBAST (PDB ID: 8JY0) with other aptamers targeting rhodamine-based derivates or structurally relevant aptamer (i. e. DIR2s, PDB ID: 6DB8), the binding interfaces between aptamers and components of ligands are also calculated. The components of ligands are indicated in the parenthesis.

Supplementary Table 3. Statistics of contact number or centroid distance between fluorophore and quencher for each group of conformation free or bound form.

Group[#]	Q_{xq}	Q_{pq}	Q_{tq}	d_{xq}[&]	d_{pq}[&]	P_{xq}[*]	P_{pq}[*]	P_{tq}[*]
1	0.1 ± 0.4	0.2 ± 0.7	0.3 ± 0.9	14.3 ± 2.7	11.3 ± 2.1	0.00	0.00	0.00
	0.0 ± 0.2	0.1 ± 0.6	0. 2 ± 0.7	14.4 ± 2.1	11.8 ± 1.6	0.00	0.00	0.00
2	33.6 ± 23.8	15.3 ± 11.9	48.9 ± 18.9	5.9 ± 1.3	5.4 ± 0.9	0.75	0.72	0.90
	8.5 ± 10.6	27.8 ± 12.6	36.3 ± 14.5	7.0 ± 0.8	4.8 ± 1.1	0.24	0.56	0.59
2.1	22.1 ± 17.9	18.8 ± 11.9	40.9 ± 15.6	6.4 ± 1.3	5.1 ± 0.8	0.44	0.51	0.58
	8.0±10.0	27.1±12.4	35.2±14.6	7.0±0.8	4.9±1.1	0.21	0.41	0.49
2.2	58.6 ± 13.0	6.7 ± 4.0	65.3 ± 14.1	4.7 ± 0.7	5.9 ± 0.5	0.29	0.18	0.29
	41.3 ± 8.2	6.2 ± 4.0	47.5 ± 9.6	5.7 ± 0.3	6.4 ± 0.5	0.01	0.00	0.01
2.3	8.6 ± 6.1	35.3 ± 8.8	43.9 ± 11.0	6.6 ± 0.4	3.9 ± 0.2	0.02	0.01	0.02
	6.1 ± 5.0	37.2 ± 7.6	43.3 ± 10.5	6.7 ± 0.3	3.9 ± 0.2	0.03	0.06	0.06

#The ensemble of TMR-DN in free- or bound-form can be roughly divided into two groups including non-contact (1) and contact (2). The latter can be further classified into 3 subgroups including contact-unstacked (2.1), stackX (2.2) and stackP (2.3).

For each group, the values from free and bound-form are positioned first and second line in each panel.

All values except possibility are represented as mean ± sd.

&The distances are represented in Å.

*The possibility P_Y is defined as ratio of number of conformers with contact number of $Q_Y \geq 5$ to the total.

Supplementary Table 4. Relative energies of the representative TMR-DN conformers with respect to the extended one.

Conformer types	Relative energy (kcal/mol)		
	r2scan-3c[#]	PWPB95-D3[@]	ωB97X-2-D3[@]
Extended	0.0 (0.0)	0.0	0.0
Bended	-5.61 (-4.42)	-4.74	-5.28
Contact-unstacked	-13.16 (-10.70)	-15.61	-17.34
StackP	-14.39 (-11.84)	-17.33	-18.30
StackX-2	-14.03 (-11.63)	-17.07	-17.37
StackX-1	-15.19 (-12.77)	-17.83	-18.85

[#]Geometrical optimizations were carried out with CPCM implicit solvent model. The enthalpy values (T = 298.15 K) were obtained through frequency calculation, which were indicated in parentheses.

[@]Single point energies were obtained using double hybrid functionals with def2-QZVPP basis set in SMD implicit solvent model.

The coordinates for each conformer along with associated energies are provided in Supplementary Data 1.

Supplementary Table 5. Thermodynamic parameters of TMR-DN binding to RhoBAST and its mutants in the presence of 10 mM Mg^{2+} as determined by ITC.

Mutation sites	**RNA**	**ΔH (kcal/mol)**	**-TΔS (kcal/mol)**	**ΔG (kcal/mol)**	***n***	***K***
PK	WT	-12.10	1.96	-10.20	1.16	34.8
	C52G	n.d.	n.d.	n.d.	n.d.	n.d.
	G35A	n.d.	n.d.	n.d.	n.d.	n.d.
	C52A	-17.60	9.71	-7.91	1.74	1600
	G31C	n.d.	n.d.	n.d.	n.d.	Weak binding
	G35C+C52G	n.d.	n.d.	n.d.	n.d.	n.d.
Interface and junction	C8G	-20.00	9.99	-10.00	1.03	47
	G37A	-18.30	8.66	-9.66	1.09	83
	A36U	-15.90	6.44	-9.43	0.91	122
	A36C	-16.80	8.79	-7.89	0.36	1400
	U30C+A36C	-27.80	19.60	-8.20	0.21	976
	ΔU7	-15.50	5.65	-9.89	1.43	56.9
	A33G	-15.40	5.27	-10.20	1.12	35.1
	A33C	-15.80	6.04	-9.80	1.31	65.5
Capping loop	G47C	n.d.	n.d.	n.d.	n.d.	Weak binding
	G47A	-5.97	-2.26	-8.23	0.64	931
	G46A	-17.10	7.99	9.09	1.26	217
	U49A	-33.60	26.20	-7.44	2.51	3510
	U48C	-13.30	3.30	-10.00	1.27	45.5
Base triple	A50U	-9.48	2.05	-7.43	0.93	3590
	A51U	-10.70	2.52	-8.15	1.37	1060

n: binding stoichiometry
n.d.: not determined

Supplementary Table 6. Basic structural parameters for RhoBAST in the presence of various [Mg^{2+}] and TMR-DN by SAXS.

RNA	Mg^{2+} (mM)	$^{a}R_g$	$^{b}R_g$	D_{max}	^{c}MW (kDa)	^{d}MW (kDa)
**RhoBAST**	0	20.66 ± 0.90	22.34 ± 0.22	80	18.83	16.95
	0.5	19.85 ± 0.52	20.6 ± 0.15	77	22.17	
	1	19.07 ± 0.27	19.44 ± 0.28	76	21.53	
	2	19.06 ± 0.62	19.38 ± 0.28	75	17.16	
	5	19.16 ± 0.29	19.23 ± 0.14	72	18.66	
	10	19.20 ± 0.55	19.03 ± 0.12	69	19.61	
	TMR-DN	19.19 ± 0.43	19.13 ± 0.12	72	19.86	

[a]derived from Guinier fitting;
[b]derived from GNOM analysis;
[c]MW: molecular weight calculated based on the power law of volume of correlation;
[d]MW: molecular weight predicted from RNA sequence.

Supplementary Table 7. Thermodynamic parameters of TMR-DN binding to RhoBAST at different [Mg^{2+}] as determined by ITC.

Mg^{2+} (mM)	ΔH (kcal/mol)	-TΔS (kcal/mol)	ΔG (kcal/mol)	*n*	K_D (nM)
0	n.d.	n.d.	n.d.	n.d.	Weak binding
0.01	-34.8	26.9	-7.83	1.7	1800
0.5	-16.4	6.01	-10.4	1.0	24.1
1	-21.8	11.4	-10.4	0.8	25.4
2	-13.4	3.10	-10.3	1.3	27.6
5	-15.7	5.49	-10.3	1.2	30.6
10	-12.1	1.96	-10.2	1.2	34.8

n: binding stoichiometry
n.d.: not determined

Supplementary Table 8. Statistics on the G4-motif in available high-resolution structures of FLAPs.

Index	Aptamer name	PDB	Containing G4 fold
1	MG	1F1T[31]	no
2	Spinach	4KZD[33]	yes
3	iSpinach	5OB3[35]	yes
4	Corn	5BJO[34]	yes
5	Squash	7KVU[41]	no
6	Chili	7OAW[42]	yes
7	Beetroot	8EYU[43]	yes
8	MangoI	5V3F[36]	yes
9	MangoII	6C63[37]	yes
10	MangoIII	6E8S[38]	yes
11	MangoIII.1	6E8T[38]	yes
12	iMangoIII	6E8U[38]	yes
13	MangoIV	6V9B[44]	yes
14	Pepper	7EOK[40]	no
15	DIR2s	6DB8[32]	no
16	TMR3	6GZK[30]	no

Supplementary Table 9. Thermodynamic parameters of RhoBAST and MG binding to their cognate ligands.

	**Mg^{2+} (mM)**	**ΔH (kcal/mol)**	**-TΔS (kcal/mol)**
RhoBAST	10	-12.1	1.96
MG	10	-17.3	7.6

Supplementary Table 10. The natural and unnatural oligonucleotide primers used in this study.

RNA	Sequence
**General**	
RhoBAST	GGAACCUCCGCGAAAGCGGUGAAGGAGAGGCGCAAGGUUAACCGCCUCAGGUUCC
RhoBAST-U1A Loop	GGAACCUCCGCCCAUUGCACUCCGGGCGGUGAAGGAGAGGCGCAAGGUUAACCGCCUCAGGUUCC
**Mutants**	
G31C	GGAACCTCCGCGAAAGCGGUCAAGGAGAGGCGCAAGGUUAACCGCCUCAGGUUCC
G35A	GGAACCTCCGCGAAAGCGGUGAAGAAGAGGCGCAAGGUUAACCGCCUCAGGUUCC
C52A	GGAACCTCCGCGAAAGCGGUGAAGGAGAGGCGCAAGGUUAAACGCCUCAGGUUCC
C52G	GGAACCTCCGCGAAAGCGGUGAAGGAGAGGCGCAAGGUUAAGCGCCUCAGGUUCC
C8G	GGAACCUGCGCGAAAGCGGUGAAGGAGAGGCGCAAGGUUAACCGCCUCAGGUUCC
G37A	GGAACCUCCGCGAAAGCGGUGAAGGAAAGGCGCAAGGUUAACCGCCUCAGGUUCC
A36U	GGAACCUCCGCGAAAGCGGUGAAGGUGAGGCGCAAGGUUAACCGCCUCAGGUUCC
A36C	GGAACCUCCGCGAAAGCGGUGAAGGCGAGGCGCAAGGUUAACCGCCUCAGGUUCC
U30C+A36C	GGAACCUCCGCGAAAGCGGCGAAGGCGAGGCGCAAGGUUAACCGCCUCAGGUUCC
G47C	GGAACCUCCGCGAAAGCGGUGAAGGAGAGGCGCAAGCUUAACCGCCUCAGGUUCC
G47A	GGAACCUCCGCGAAAGCGGUGAAGGAGAGGCGCAAGAUUAACCGCCUCAGGUUCC
G46A	GGAACCUCCGCGAAAGCGGUGAAGGAGAGGCGCAAAGUUAACCGCCUCAGGUUCC
U49A	GGAACCUCCGCGAAAGCGGUGAAGGAGAGGCGCAAGGUAAACCGCCUCAGGUUCC
A50U	GGAACCUCCGCGAAAGCGGUGAAGGAGAGGCGCAAGGUUUACCGCCUCAGGUUCC
A51U	GGAACCUCCGCGAAAGCGGUGAAGGAGAGGCGCAAGGUUAUCCGCCUCAGGUUCC
U48C	GGAACCUCCGCGAAAGCGGUGAAGGAGAGGCGCAAGGCUAACCGCCUCAGGUUCC
A33G	GGAACCUCCGCGAAAGCGGUGAGGGAGAGGCGCAAGGUUAACCGCCUCAGGUUCC
A33C	GGAACCUCCGCGAAAGCGGUGACGGAGAGGCGCAAGGUUAACCGCCUCAGGUUCC
ΔU7	GGAACCCCGCGAAAGCGGUGAAGGAGAGGCGCAAGGUUAACCGCCUCAGGUUCC
C52G+G35C	GGAACCTCCGCGAAAGCGGUGAAGCAGAGGCGCAAGGUUAAGCGCCUCAGGUUCC

Supplementary References

1. Neese, F. Software update: The ORCA program system-Version 5.0. *Wiley Interdisciplinary Reviews-Computational Molecular Science* **12**, e1606 (2022).
2. Klamt, A. Conductor-like screening model for real solvents - a new approach to the quantitative calculation of solvation phenomena. *Journal of Physical Chemistry* **99**, 2224-2235 (1995).
3. Grimme, S., Hansen, A., Ehlert, S. & Mewes, J.M. r(2)SCAN-3c: A "Swiss army knife" composite electronic-structure method. *Journal of Chemical Physics* **154**, 064103 (2021).
4. Marenich, A.V., Cramer, C.J. & Truhlar, D.G. Universal solvation model based on solute electron density and on a continuum model of the solvent defined by the bulk dielectric constant and atomic surface tensions. *Journal of Physical Chemistry B* **113**, 6378-6396 (2009).
5. Goerigk, L. & Grimme, S. Efficient and accurate double-hybrid-meta-GGA density functionals-evaluation with the extended gmtkn30 database for general main group thermochemistry, kinetics, and noncovalent interactions. *Journal of Chemical Theory and Computation* **7**, 291-309 (2011).
6. Chai, J.D. & Head-Gordon, M. Long-range corrected double-hybrid density functionals. *Journal of Chemical Physics* **131**, 174105 (2009).
7. Schauperl, M., Nerenberg P.S., Jang H., Wang L.P., Bayly C.I., Mobley D.L. & Gilson M.K. Non-bonded force field model with advanced restrained electrostatic potential charges (RESP2). *Communications Chemistry* **3**, 1-11 (2020).
8. Weigend, F., Furche, F. & Ahlrichs, R. Gaussian basis sets of quadruple zeta valence quality for atoms H-Kr. *Journal of Chemical Physics* **119**, 12753-12762 (2003).
9. Perdew, J.P., Ernzerhof, M. & Burke, K. Rationale for mixing exact exchange with density functional approximations. *Journal of Chemical Physics* **105**, 9982-9985 (1996).
10. Zheng, J., Xu, X. & Truhlar, D.G. Minimally augmented Karlsruhe basis sets. *Theoretical Chemistry Accounts* **128**, 295-305 (2011).
11. Bayly, C.I., Cieplak, P., Cornell, W.D. & Kollman, P.A. A well-behaved electrostatic potential based method using charge restraints for deriving atomic charges - the resp model. *Journal of Physical Chemistry* **97**, 10269-10280 (1993).
12. He, X.B., Man, V.H., Yang, W., Lee, T.S. & Wang, J.M. A fast and high-quality charge model for the next generation general AMBER force field. *Journal of Chemical Physics* **153**, 114502 (2020).
13. Perez, A., Marchan, I., Svozil, D., Sponer, J., Cheatham, T.E., Laughton, C.A. & Orozco, M. Refinenement of the AMBER force field for nucleic acids: improving the description of alpha/gamma conformers. *Biophysical Journal* **92**, 3817-3829 (2007).
14. Steinbrecher, T., Latzer, J. & Case, D.A. Revised AMBER parameters for bioorganic phosphates. *Journal of Chemical Theory and Computation* **8**, 4405-4412 (2012).
15. Zgarbova, M., Otyepka, M., Spone,r J., Mládek, A., Banáš, P., Cheatham, T.E., Jurečka, P. Refinement of the Cornell et al. Nucleic acids force field based on reference quantum chemical calculations of glycosidic torsion profiles. *Journal of Chemical Theory and Computation* **7**, 2886-2902 (2011).
16. Cheatham, T.E., Cieplak, P. & Kollman, P.A. A modified version of the Cornell et al. Force field with improved sugar pucker phases and helical repeat. *Journal of Biomolecular Structure & Dynamics* **16**, 845-862 (1999).
17. Cornell, W.D., Cieplak, P., Bayly, C.I., Gould, I.R. A second generation force field for the simulation of proteins, nucleic acids, and organic molecules. *Journal of the American Chemical Society* **118**, 2309-2309 (1996).
18. Izadi, S., Anandakrishnan, R. & Onufriev, A.V. Building water models: a different approach. *Journal of Physical Chemistry Letters* **5**, 3863-3871 (2014).
19. Sengupta, A., Li, Z., Song, L.F., Li, P.F. & Merz, K.M. Parameterization of monovalent Ions for the OPC3, OPC, TIP3P-FB, and TIP4P-FB water models. *Journal of Chemical Information and Modeling* **61**, 3734-3735 (2021).
20. Tribello, G.A., Bonomi, M., Branduardi, D., Camilloni, C. & Bussi, G. PLUMED 2: new feathers for an old bird. *Computer Physics Communications* **185**, 604-613 (2014).
21. Abraham, M.J., Murtola, T., Schulz, R., Szilárd, P., Smith, J.C., & Hess, B. GROMACS: High performance molecular simulations through multi-level parallelism from laptops to

supercomputers. *SoftwareX* **1-2**, 19-25 (2015).
22. Rahman, A. Polymorphic transitions in single crystals: a new molecular dynamics method. *Journal of Applied Physics* **52**, 7182 (1981).
23. Bussi, G., Donadio, D. & Parrinello, M. Canonical sampling through velocity rescaling. *The Journal of Chemical Physics* **126**, 014101 (2007).
24. Hess, B., Bekker, H., Berendsen, H.J.C. & Fraaije, J.G.E.M. LINCS: A Linear Constraint Solver for molecular simulations. *Journal of Computational Chemistry* **18**, 1463-1472 (1998).
25. Darden, T., York, D. & Pedersen, L. Particle mesh Ewald: An Nlog(N) method for Ewald sums in large systems. *Journal of Chemical Physics* **98**, 10089-10092 (1993).
26. Appadurai, R., Nagesh, J. & Srivastava, A. High resolution ensemble description of metamorphic and intrinsically disordered proteins using an efficient hybrid parallel tempering scheme. *Nature Communications* **12**, 958 (2021).
27. Humphrey, W., Dalke, A. & Schulten, K. VMD: Visual molecular dynamics. *Journal of Molecular Graphics & Modelling* **14**, 33-38 (1996).
28. Best, R.B., Hummer, G. & Eaton, W.A. Native contacts determine protein folding mechanisms in atomistic simulations. *Proceedings of the National Academy of Sciences of the United States of America* **110**, 17874-17879 (2013).
29. Gabb, H.A., Sanghani, S.R., Robert, C.H. & Prevost, C. Finding and visualizing nucleic acid base stacking. *Journal of Molecular Graphics & Modelling* **14**, 6-11 (1996).
30. Elke, D. F., Michael, J., Benjamin, B., Tobias, M., Christoph, K., & Ohlenschläger Oliver. Structure of an RNA aptamer in complex with the fluorophore tetramethylrhodamine. *Nucleic Acids Research* **48**, 949-961 (2020).
31. Baugh, C., Grate, D. & Wilson, C. 2.8 Å crystal structure of the malachite green aptamer. *Journal of Molecular Biology* **301**, 117-128 (2000).
32. Shelke, S.A., Shao, Y., Laski, A., Koirala, D., Weissman, B.P., & Fuller, J.R. Structural basis for activation of fluorogenic dyes by an RNA aptamer lacking a G-quadruplex motif. *Nature Communications* **9**, 4542 (2018).
33. Huang, H., Suslov, N. B., Li, N.S., Shelke, S.A., Evans, M.E., Koldobskaya, Y., Rice, P.A., & Piccirilli J.A. A G-quadruplex – containing RNA activates fluorescence in a GFP-like fluorophore. *Nature Chemical Biology* **10**, 686-691 (2014).
34. Warner, K. D., Sjekloa, L., Song, W., Filonov, G. S., Jaffrey, S. R., & Adrian R Ferré-D'Amaré. A homodimer interface without base pairs in an RNA mimic of red fluorescent protein. *Nature Chemical Biology* **13**, 1195-1201 (2017).
35. Fernandez-Millan, P., Autour, A., Ennifar, E., Westhof, E. & Ryckelynck, M. Crystal structure and fluorescence properties of the iSpinach aptamer in complex with DFHBI. *RNA* **23**, 1788-1795 (2017).
36. Trachman, R.J., Demeshkina, N.A., Lau M.W.L., Panchapakesan S.S.S., Jeng S.C.Y., Unrau P.J. & Adrian R Ferré-D'Amaré. Structural basis for high-affinity fluorophore binding and activation by RNA Mango. *Nature Chemical Biology* **13**, 807-813 (2017).
37. Trachman, R.J., Abdolahzadeh A., Andreoni A., Cojocaru R., Knutson J.R., Ryckelynck M., Unrau P.J. & Adrian R Ferré-D'Amaré. Crystal structures of the Mango-II RNA aptamer reveal heterogeneous fluorophore binding and guide engineering of variants with improved selectivity and brightness. *Biochemistry* **57**, 3544-3548 (2018).
38. Trachman, R.J., Autour, A., Jeng, S.C.Y., Abdolahzadeh, A., Andreoni, A., & Cojocaru, R. Structure and functional reselection of the Mango-III fluorogenic RNA aptamer. *Nature Chemical Biology* **15**, 472-479 (2019).
39. Jeng, S.C.Y., Trachman, R.J., Weissenboeck, F., Truong, L., Link, K.A., & Jepsen, M.D.E. Fluorogenic aptamers resolve the flexibility of RNA junctions using orientation-dependent FRET. *RNA* **27**, 433-444 (2021).
40. Huang, K., Cheng X., Li, C., Song, Q., Li, H., Zhu, L., Yang, Y. & Ren, A. Structure-based investigation of fluorogenic Pepper aptamer. *Nature Chemical Biology* **17**, 1289-1295 (2021).
41. Truong, L., Kooshapur, H., Dey, S.K., Li, X., Tjandra, N., Jaffrey, S.R. & Adrian R Ferré-D'Amaré. The fluorescent aptamer Squash extensively repurposes the adenine riboswitch fold. *Nature Chemical Biology* **18**, 191-198 (2022).

42. Mieczkowski, M., Steinmetzger, C., Irene Bessi, I., Ann-Kathrin Lenz, A.K. & Hbartner, C. Large stokes shift fluorescence activation in an RNA aptamer by intermolecular proton transfer to guanine. *Nature Communications* **12**, 3549 (2021).
43. Passalacqua, L.F.M., Starich, M.R., Link, K.A., Wu, J., Knutson, J.R., Tjandra, N., Jaffrey, S.R. & Adrian R Ferré-D'Amaré. Co-crystal structures of the fluorogenic aptamer Beetroot show that close homology may not predict similar RNA architecture. *Nature Communications* **14**, 2969 (2023).
44. Trachman, R.J., Cojocaru, R., Wu, D., Piszczek, G. & Adrian R Ferré-D'Amaré. Structure-guided engineering of the homodimeric Mango-IV fluorescence turn-on aptamer yields an RNA FRET pair. *Structure* **28**, 776-785 (2020).
