# Supplementary material for: Structural mechanisms for binding and activation of a contact-quenched fluorophore by RhoBAST
Source: Nat Commun. 2024 May 17;15:4206. doi: 10.1038/s41467-024-48478-9 (PMC11101630; doi:10.1038/s41467-024-48478-9)
Supplement: Supplementary file 3 — Description of additional supplementary files [file 41467_2024_48478_MOESM3_ESM.pdf]

<u>Description of Additional Supplementary Files</u>

Supplementary Data 1: The coordinate and energy of TMRDN, force field parameter file for TMR-DN and script for contact analysis.
